# Strength-based physical therapy for anxiety and depressive symptoms in a war-exposed adult: a case report

**DOI:** 10.1093/omcr/omag098

**Published:** 2026-06-15

**Authors:** Volodymyr Maksym, Vira Budzyn, Olena Khanikiants

**Affiliations:** Department of Physical Therapy and Sports Medicine, Lviv State University of Physical Culture named after Ivan Boberskyi, 8 Dudayeva St., Lviv 79005; Kostiushka St. 11, Lviv 79007, Ukraine; Department of Physical Therapy and Sports Medicine, Lviv State University of Physical Culture named after Ivan Boberskyi, 8 Dudayeva St., Lviv 79005; Kostiushka St. 11, Lviv 79007, Ukraine; Department of Theory and Methods of Physical Education, Lviv State University of Physical Culture named after Ivan Boberskyi, 11 Kostiushka St., Lviv, Lviv Oblast 79007, Ukraine

**Keywords:** resistance training, physical therapy, anxiety, depression, insomnia, chronic pain

## Abstract

**Background:**

Anxiety and depressive symptoms are common in war-exposed populations. Resistance exercise shows growing evidence for mental health outcomes, but practical implementation details are often underreported.

**Case:**

A 32-year-old male in a civilian period after military service reported 6 months of severe anxiety (7/10) with poor tolerance of crowded places, depressive symptoms (7/10), insomnia requiring diphenhydramine, tremor, poor appetite and widespread pain. Baseline Beck Depression Inventory-II (BDI-II) and Beck Anxiety Inventory (BAI) scores were 27 and 30, respectively.

**Intervention:**

A fully supervised, gym-based, strength-oriented physical therapy programme was delivered three times weekly for ~ 6 months using patient-centred autoregulation, mixed repetition ranges and readiness-guided rest.

**Outcome:**

At ~ 6 months, BDI-II decreased to 7 and BAI to 8; sleep normalised, pain resolved and social functioning improved. No recurrence was reported at ~ 12 months.

**Conclusion:**

This case supports the feasibility of patient-centred resistance-based physical therapy as an adjunct within multidisciplinary care.

## Introduction

Case reports are valuable for showing ‘what was done’ in real-world care, especially when complex symptoms and context make controlled conditions difficult to replicate, and they benefit from transparent reporting standards [1]. Resistance training has been associated with reductions in anxiety and depressive symptoms in randomised trials and meta-analyses, but translation into routine rehabilitation still benefits from clear, practical descriptions of supervision, autoregulation and progression [2, 3]. We report a de-identified case of marked improvement in anxiety and depressive symptoms during a structured, strength-based physical therapy programme.

## Case report

### Patient information and presentation

A 32-year-old male (176 cm, 95 kg) was assessed during a post-deployment civilian period. He had served for several years, including deployment during active hostilities; symptoms began within months of return and persisted for approximately 6 months prior to programme initiation. He reported 6 months of severe anxiety (7/10) with strong discomfort in crowded places, irritability and reliance on music via headphones to cope. Depressive symptoms were rated 7/10 with apathy ~ 5/10. Sleep onset required diphenhydramine. He reported chronic widespread pain (back, shoulders, legs and headaches), tremor, poor appetite and mood swings.

Pain intensity was not prospectively recorded with a numeric rating scale (NRS). Retrospectively, based on functional limitation (needing ~ 20–30 minutes to get out of bed due to pain), the patient described baseline pain as approximately 8/10, improving to ~ 0/10 by follow-up.

The patient had served as a combat medic during active hostilities, reporting repeated exposure to traumatic events and sustained operational stressors. To protect confidentiality, specific locations, unit information, and event details are not reported. He also reported multiple service-related injuries, including 3 concussions.

Social history: the patient was in a relationship during follow-up and reported having a supportive partner; he reported no children.

### Medical history and screening

History included multiple injuries, a road traffic accident and 2–3 concussions. He reported alcohol, nicotine and caffeine use. Physical activity prior to the programme was intermittent gym training. A PAR-Q/clinical screen identified no contraindications to exercise participation.

### Medication history

At programme start (Week 0; summer 2023), the patient reported using diphenhydramine for sleep only (exact dose and frequency were not recorded). This was a short-term symptomatic sleep aid reported by the patient and was not initiated by the authors. Long-term insomnia management remained under the treating psychiatrist. He described previous psychiatric pharmacotherapy earlier in the symptom course (sertraline, pregabalin and trazodone) prescribed and adjusted by a psychiatrist; exact doses and dates were unavailable.

### Outcome measures

Symptom severity was self-assessed in Ukrainian using BDI-II and BAI at baseline and at follow-up (Table 1) [4, 5]. Key time points are summarised in Table 2.

**Table 1:** Psychological outcomes.

Assessment	BDI-II	BAI
Baseline (Week 0)	27	30
Follow-up (~Month 6)	7	8

**Table 2:** Timeline.

Time point	Key events
~6 months before Week 0	Worsening anxiety, depressive symptoms, insomnia, tremor, poor appetite, widespread pain
Week 0 (summer 2023)	Baseline: BDI-II 27; BAI 30; diphenhydramine for sleep; begins supervised strength-based PT
~Month 6 (approx.)	Follow-up: BDI-II 7; BAI 8; sleep normalised; pain absent; improved tolerance of crowded environments
~Month 12	No recurrences reported; maintained gains

### Therapeutic intervention

Session duration and format. Each supervised session lasted approximately 60–75 minutes (typically including ~ 10–15 minutes warm-up, ~ 35–45 minutes main resistance exercises, and ~ 10–15 minutes cool-down/breathing and walking). Sessions were delivered in a gym setting under continuous therapist supervision. A fully supervised, gym-based strength programme was delivered three times weekly for approximately 6 months (estimated ~ 70–80 sessions). Sessions used patient-centred autoregulation with shared decision-making around set selection, rest and progression. Refusal of a set was treated as a valid stop criterion, aiming to reduce anticipatory anxiety and support perceived control.

### Session structure

- Warm-up: joint mobility and light dynamic movements.
- Main exercises: typically organised as Pull/Push/Legs.
  - Pull: deadlift variations, lat pulldown, cable/seated row, shrugs.
  - Push: barbell bench press, incline dumbbell press, overhead press (standing), seated dumbbell press.
  - Legs/hips: leg press, hip thrust/glute bridge; machine leg extension/curl/abduction as tolerated.
- Accessory/core: dumbbell curls (hammer and supinated), triceps pushdowns, rope face pulls, lateral raises, abdominal crunch variations.
- Cool-down: light work for smaller muscle groups, walking and breathing; myofascial release and stretching when hypertonicity was noted (pectorals, gluteals, back).

### Intensity, rest and progression

Repetition ranges varied depending on readiness: occasional low-repetition heavy sets (including singles), moderate sets (e.g. 8–10) and high-repetition sets (up to ~ 25–33). Typical rest was 1–3 minutes, extended up to ~ 7 minutes for heavy singles. Progression prioritised safety: load was increased when recovery and psychological readiness were favourable; otherwise, progression used additional repetitions or sets in moderate/high repetition ranges.

### Safety

No serious adverse events were reported. Occasional muscle spasms resolved with stretching and myofascial release. No injuries were reported.

## Discussion

This case shows the practical feasibility of a supervised, strength-based physical therapy programme as an adjunct approach for anxiety and depressive symptoms in a war-exposed adult. The intervention deliberately emphasised predictability, shared control and readiness-guided rest—elements that may be particularly helpful when anxiety is high and perceived loss of control drives avoidance.

The marked reductions in BDI-II/BAI scores, alongside improvements in sleep, pain and social functioning, are consistent with evidence from meta-analyses that resistance training can reduce depressive and anxiety symptoms [2, 3]. Still, causality cannot be inferred from one case. Medication history was incomplete (and managed by a psychiatrist), and the patient experienced multiple concurrent psychosocial changes (housing, relationships, daily activity and hobbies), any of which could have contributed. Limitations also include absence of prospective pain/sleep scales and reliance on retrospective pain estimation.

Future prospective cases should include systematic measures (e.g. NRS and a brief insomnia scale) and clearer medication timelines to strengthen attribution.
